# A critical interpretive synthesis of migrants’ experiences of the Australian health system

**DOI:** 10.1186/s12939-022-01821-2

**Published:** 2023-01-09

**Authors:** Kimberly Lakin, Sumit Kane

**Affiliations:** Nossal Institute for Global Health, School of Population and Global Health, The University of Melbourne, Level 2, 32 Lincoln Square, Melbourne, 3010 Australia

**Keywords:** Migrants, Healtcare experience, Health system responsiveness, Cultural competency

## Abstract

While the health of and healthcare use by migrants has received significant scholarly and policy attention in Australia, current debates highlight that a critical examination of the theoretical underpinnings of these inquiries and responses is needed. We conducted a systematic review and critical interpretive synthesis (CIS) to critically examine how the policy and scholarly literature conceptualises migrants’ interactions with and experiences of the Australian health system. Guided by PRISMA, we searched for literature without imposing any limits. We also searched key State and Federal Government websites for relevant policy documents. Our initially broad inclusion criteria became refined as the CIS progressed. We prioritised the likely relevance and theoretical contribution of the papers to our inquiry over methodological quality. The CIS of 104 papers revealed that the Australian scholarly literature and policy documents consistently homogenise and reduce migrants according to an assumed, (1) cultural identity, (2) linguistic affiliation, and/or (3) broad geographic origin. Based on these three critiques and drawing on the theoretical literature, we propose a synthesising argument on how the Australian literature could better conceptualise migrants’ experiences of the Australian health system. We contend that both research and policy should explicitly recognise and engage with the multifaceted and shifting ways that migrants define themselves, generally, and during their encounters with destination country health systems. Engagement with this notion is necessary for also understanding how aspects of migrants’ identities are dynamically co-constructed during their interactions with the health system. These understandings have implications for improving the design and implementation of policies and programs directed at improving the responsiveness of Australia’s health system to the needs and expectations of migrant communities specifically, and destination countries broadly.

**Supplementary Information:**

The online version contains supplementary material available at 10.1186/s12939-022-01821-2.

## Background

The scale of international migration has continued to increase, with an estimated 281 million international migrants in 2020 [1]. Contemporary migration is a complex phenomenon which is driven by a range of factors such as economic pressures, political violence, and climate change [2]. The phenomenon is also a dynamic process wherein an individuals’ migration status evolves as the migration journey unfolds in time and space [3]. In view of this, there is increasing recognition of the multisector determinants of migrant health including, for example, individuals’ pre-departure health, as well as socioeconomic and environmental conditions at the origin, destination, and sometime during the journey [4]. However, globally, cultural frameworks currently dominate research on migrant health; these view culture as the primary determinant of individual health behaviours that subsequently affect health outcomes. Such explanations have long been critiqued for obscuring the effect of structural factors on migrant health outcomes [2, 3, 5]; these debates highlight that a critical examination of the theoretical underpinnings of current inquiries is needed. However, this also requires a critical analysis of the various policies and initiatives that guide and frame research and practice on migrant health.

With approximately 30% of Australia’s population born overseas [6], Australia is considered as one of the major ‘immigration nations’. While to date, there is no formal coordinated national policy targeted towards improving migrant health in Australia, most States and Territories do have policies on healthcare provision for what are called ‘Culturally and Linguistically Diverse (CALD)’ communities; the CALD moniker includes migrants and refugees. The health of and healthcare use by CALD communities has received significant scholarly attention in Australia. For instance, recently, Au et al. [7] conducted a systematic review of refugees’ perceptions of the Australian health system; similarly, Billett et al. [8] reviewed migrant and refugee women’s experiences of Australian maternity care. However, a comprehensive and critical interrogation of the literature on migrants’ experiences of the Australian health system is yet to be undertaken – to our knowledge no such work has been done in any other ‘immigration nation’ either. Engaging with current debates and knowledge gaps, we conducted a critical interpretive synthesis (CIS) to critically examine how the policy and scholarly literature conceptualises migrants’ care experiences in Australia.

This CIS was guided by the refined compass question – how could the Australian literature better conceptualise migrants’ experiences of the Australian health system? To respond to this question, we first aim to systematically and critically examine how the empirical literature on migrants’ experiences of the Australian health system, as well as State and Federal Government health policies, conceptualise and understand migrants’ experiences of care Then, we aim to examine the gaps in the current literature and draw on the broader theoretical literature, to propose how Australian researchers and policymakers can better understand and conceptualise healthcare for migrant communities, in the form of a *synthesising argument*.

## Methods

This critical interpretive synthesis is reported according to the Preferred Reporting Items for Systematic Reviews and Meta-Analyses (PRISMA) checklist (Additional File 1). While doing so, we take into account the differences between CISs and conventional systematic reviews with respect to the processes of literature search, selection, critical appraisal, and synthesis.

## Design

A critical interpretive synthesis (CIS) is an approach to systematically analyse literature with an emphasis on critical appraisal, theory development, and flexibility [9, 10]. It uses conventional systematic review processes, while incorporating qualitative inquiries, to examine both empirical and non-empirical literature. General activities which represent the dynamic process of CIS include: the formulation of an initial, ‘compass’ question (which is refined as the review progresses), literature search, selection, critical appraisal, data extraction and synthesis, and the development of an overarching *synthesising argument* [9, 10]. In this way, CIS is particularly suitable for synthesising a diverse and ambiguous body of evidence and where the primary aim of the review is *interpretive*, rather than *aggregative*. The current literature on migrants’ experiences of the Australian health system is diverse, both in terms of the type of health services and migrant communities examined. The flexibility and critical orientation associated with the CIS approach to systematically review literature therefore makes it a suitable approach to understand how current Australian literature has, and could better, conceptualise migrants’ experiences of care. As a CIS enables the analysis of both empirical and non-empirical literature, it is therefore also possible to critically examine Australian policies and initiatives which inform current research on migrants’ encounters with the Australian heath system. The CIS approach has been effectively used to systematically and critically review the literature on health systems processes, and the roles and interactions of key health system actors [11–13].

## Literature search

**Fig. 1:**
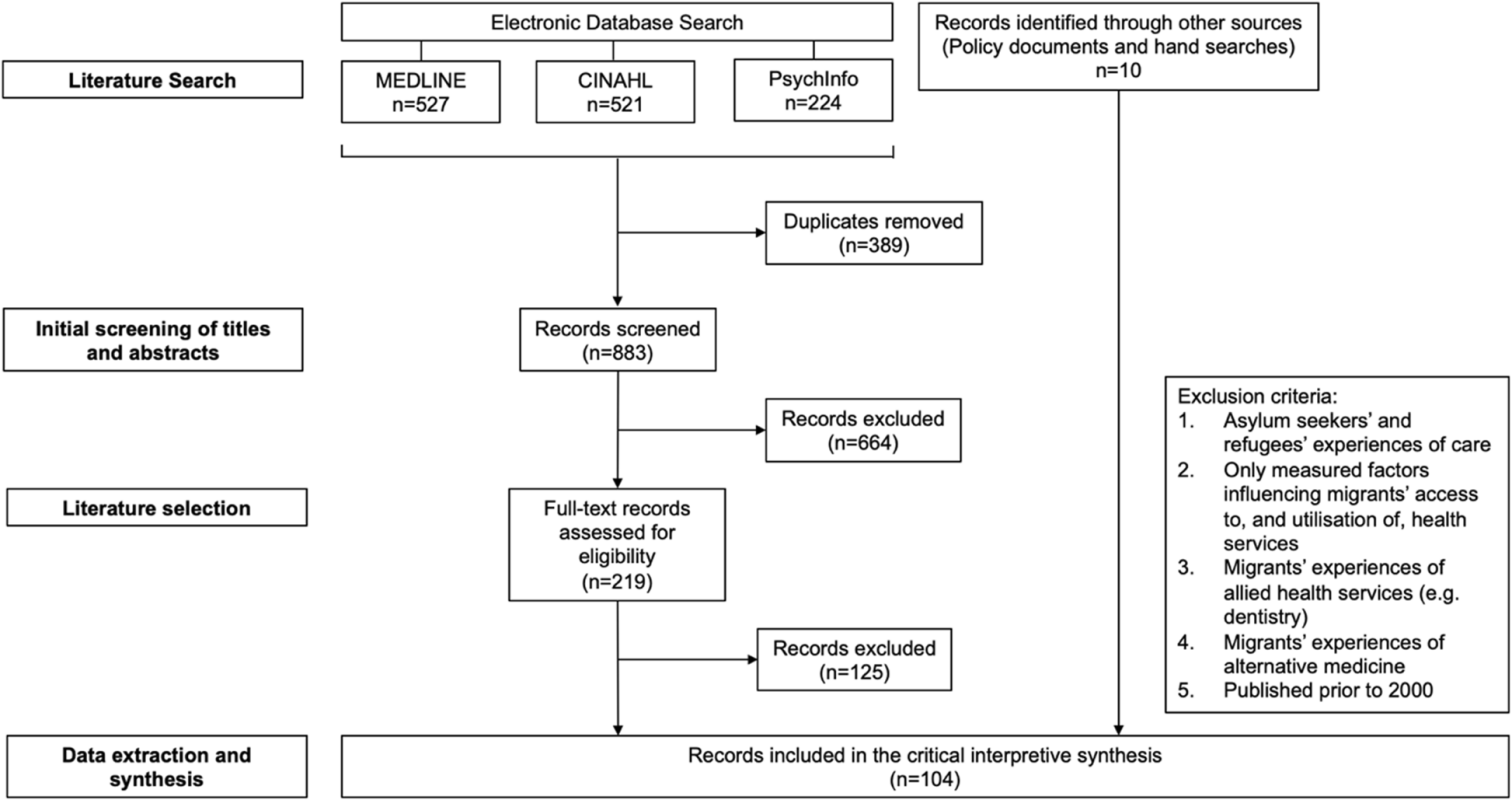
Literature search and selection

The first stage involved a search of electronic bibliographic databases, as outlined in Fig. 1. Based on our initial readings and understandings, keywords were identified, and appropriate bibliographic databases were selected. Initially, we searched broadly in MEDLINE, CINAHL and PsychInfo databases from February to March 2021 using the terms: (migrant*, immigrant*) AND (health system*, health care, health service*, health-care, healthcare) AND (Australia*, Victoria, Northern Territory, New South Wales, Tasmania, Queensland). [9, 10]. In parallel with the search of bibliographic databases, additional records were identified by hand searching the reference lists of key publications and systematic reviews. Additionally, we decided to include relevant State and Federal Government health policies in this review to understand how the Australian policy landscape has potentially shaped conceptualisations of the migrant care experience in current empirical literature.

Therefore, websites of Departments of Health and Human Services of State Governments, and the Australian Commonwealth Government’s Department of Health were also hand searched.

## Literature selection

The initial titles and abstracts of records captured by the literature search were screened for inclusion. An Endnote database was developed to manage the search results; duplicates were removed, and the titles and abstracts of each article were screened. During this process, we erred on the side of inclusivity and broadly included studies that examined migrants’ experiences with various aspects of the Australian health system. Therefore, we did not exclude any specific type of health service that migrant patients engaged with. However, papers that, (1) solely focused on the care experiences of asylum seekers and refugees, (2) narrowly focused on measuring factors influencing migrants’ access to and, utilisation of, health services, and did not shed light on the complexity of the migrant care experience, (3) examined migrants’ experiences of allied health services (such as dentistry) and, (4) focused on alternative medicine, were excluded. Articles which we were uncertain about inclusion/exclusion were set aside and their relevance to the emerging inquiry and compass question was discussed.

Full-text records of included studies were retrieved and assessed for eligibility and quality (detailed further below). Full-text records of eligible studies were retrieved and analysed in sets of 50 so that data selection and synthesis could occur concurrently. However, as part of the early assessments of full-text records, nine further survey-only studies that narrowly focused on measuring factors influencing migrants’ access to and, utilisation of, health services were identified, and were thus excluded. Moreover, ideas around ‘cultural competence’ gained traction and prominence in the Australian social and health policy orientations in the late nineties and early 2000s. During this period, ideas around cultural competence became firmly embedded within Australian professional standards for all medical professionals and started getting incorporated into various significant Australian health policy documents [14]. We therefore decided to also limit the search to empirical literature from after the year 2000. Early assessments of records enabled us to modify and refine the compass question, as well as to begin formulating our emerging critique of the literature.

## Critical appraisal

Our judgements about the quality of included papers were informed by Dixon-Woods et al.’s [9] appraisal prompts in a two-step process. During this process, we prioritised the likely relevance (“signal”) and theoretical contribution of the paper to our inquiry, over methodological quality (“noise”) [9, 10]. In step one, prior to data extraction and synthesis, the appraisal prompts were used to assess the methodological quality of each paper and “fatally flawed” papers were excluded from the synthesis. Finally, in step 2 during synthesis, we assessed the relevance of the paper and those that did not provide valuable insights to the CIS were excluded.

## Data extraction and synthesis

A data extraction proforma was developed to identify notable characteristics of each paper (see Additional file 2). Included papers were sorted and organised using QSR NVivo. During the process of synthesis, each paper was read several times to firstly identify emerging themes and the relationships between them. Notable patterns that emerged from the data were used as “triggers” for exploring relevant theoretical literature on how the “migrant” has been conceptualised and understood in the global literature. Our three critiques of the Australian scholarly literature (detailed below) were then developed, informed by the emergent themes and the relevant theoretical literature. Consistent with the methodology of a CIS, the process of synthesis then culminated in the development of our synthesising argument on how Australian literature and policy could better conceptualise migrants’ care experiences.

To firstly understand how migrants’ experiences of the Australian health system have been conceptualised in the current empirical literature, we examined work by Bourdieu on language and culture [15], as well as Weber’s work on language, ethnicity and the nation-state [16]. Current thinking on cultural safety, cultural competence and cultural responsiveness also informed our interpretations of the Australian health policies and initiatives we included in the review [14, 17, 18]. Finally, to develop our synthesising argument on how the Australian scholarly literature could better conceptualise migrants’ care encounters, we drew on literature on the multiplicity and fluidity of the “migrant identity” by Li and Findlay [19], McAreavey [20], Hack-Polay et al. [21] and Brah [22]. We also closely examined work by Anthias [23], Pedwell [24], Hill [25] and Carter and Mireles [26] to better understand the notion of “groupism”, as well as the intersectional nature of identity constructs. The links between the empirical evidence and the theoretical literature we drew upon in this review is outlined in greater detail in Additional file 3.

Therefore, our approach to the process of synthesis was slightly different from the inductive approach that Dixon-Woods and colleagues [9] have proposed. The essential task of interpretive reviews, they suggest, is the development of theory grounded in the studies included in the review [9]. However, Tavory and Timmermans [27] put forth an abductive analytical approach which aims to develop new theorisations based on identifying surprising evidence that does not fit within existing theoretical understandings. They argue that one needs to be “theoretically sensitised” in order to develop novel understandings; an in-depth knowledge of theoretical underpinnings of a phenomenon is necessary to identify surprising observations and to stimulate new understandings [27]. In view of this, we took an abductive approach to synthesis instead. Depraetere et al. [10] have noted that many published CISs have not taken a purely inductive approach; some have either adopted a deductive approach with theories applied to the data, or a combination of both inductive and deductive analytical approaches.

## Results

### Search results and article selection

A total of 104 papers were included in our critical interpretive synthesis of migrants’ experiences of the Australian health system (see Fig. 1). The electronic bibliographic database search yielded 1272 records and, after the removal of duplicates (*n* = 389), the titles and abstracts of 883 records were screened. Based on our initial board exclusion criteria, 664 records were excluded. Following further refinement of the exclusion criteria and additional searches, 104 papers were included in the CIS. Details of the included papers and policy documents are presented in Additional file 2.

### How are migrants’ experiences of the Australian health system conceptualised?

We began this CIS by first aiming to examine how the literature conceptualised migrants’ experiences of the Australian health system. This was with a view to ultimately propose how the Australian literature could better conceptualise migrants’ care encounters. In many papers, the migrant patient was reduced to their “cultural identity” and this led to an overwhelming focus on how culture shapes migrants’ care encounters. Other papers reduced migrant patients to specific language groups, conveying a sense of “linguistic homogeneity” and emphasising the impact of language barriers on the care experience. Finally, some studies reduced and homogenised migrants’ geographic origins, discounting the diversity and heterogeneity within and across regions and countries.

In the following sections we present our critique of the Australian literature on migrants’ care along these three broad lines which represent the key findings of our CIS. Additional file 3 presents a detailed account of how these three lines of critique were identified and arrived at, and how the overall synthesising argument was developed – it provides the empirical and theoretical basis of each critique.

### Critique 1: Reduction of the migrant patient to ‘a’ cultural identity

The influence of culture on migrants’ experiences of the Australian health system was widely reported and discussed in the literature. Cultural mores, beliefs, and the stigma associated with these beliefs, influenced various aspects of migrants’ care encounters. In particular, culture shaped migrant patients’ decisions and ability to access care, their trust in healthcare providers (as well as interpreters), and their need for confidentiality during the healthcare encounter. This was particularly the case when accessing health services for mental health and sexual and reproductive health – this being linked to a preference among migrant patients for being treated by culturally and linguistically concordant healthcare practitioners. However, we noted that in articulating these influences, migrant patients were often homogenised and reduced to broad cultural groups. For instance, Alzubaidi et al. [28], suggest that “collectivistic Arabic cultures” can have some “problematic aspects”, specifically, that significant others are often involved in decision-making processes, including those related to health. In another study, it was found that Asian migrant women were at times reluctant to express their childbirth-related needs or preferences. When Hoang et al. (p.7, [29]) explain the nature of doctor-patient interactions they contend that “most Asian cultures teach people to be unassertive and inhibited from childhood”. In examining the health needs of migrant mothers from different cultural backgrounds, Renzaho and Oldroyd [30] report that Chinese, Middle Eastern and African mothers, more so than Afghani mothers, experienced differences between their traditional antenatal practices and those in Australia. Within these studies, the assumption is that migrant patients view themselves as “members” of specific cultural groups and therefore adhere to the common practices and beliefs of these groups [31]. In this way, categorising migrant patients according to their cultural identity led to an inordinate focus on ‘a’ culture as the key factor shaping migrants’ experiences of the Australian health system. However, there were exceptions which recognised the limitations of such a culturalist approach and appreciated the importance of considering the “individuality” of migrant patients. For example, in Broom et al.’s [32] study, healthcare practitioners described culture as “part of the complexity” and emphasised the need to manage the “reduction of personhood to cultural abstractions”.

Given the above, it was therefore unsurprising that Australian policies on migrant health were also framed within a broad, cultural frame – which paradoxically was rather narrow. Healthcare policies across the board recognised and emphasised that culture, in particular, influences how individuals experience and manage health and illness [33, 34]. The “cultural responsiveness framework” has emerged as the guiding principle for Australian health services to provide care that is “respectful of, and relevant to, the health beliefs, health practices, culture and linguistic needs” of diverse communities [35]. Recent health policy documents tend to argue and demur that ‘cultural responsiveness’ should be understood as being a broader concept than ‘cultural competence’ (this was the focus of policies before 2005) as it better encompasses the diverse needs of migrant populations [34]. However, despite this claim, we found that definitions of cultural responsiveness, within multiple Australian health policies, were centred predominantly on addressing some particular, almost titular, cultural, and linguistic needs of migrant communities [33–36]. In fact, several studies highlighted the shortcomings of the cultural competence/responsiveness premise within healthcare policies. For instance, in the study by Broom et al. [32], one healthcare professional confessed, “I’ve come across too many cultures to be competent in all of them…”. This reflects a common feeling among healthcare professionals of being overwhelmed and unprepared for encounters with patients from diverse backgrounds which was exacerbated by a lack of cross-cultural training [37–39].

These findings echo current debates within the broader global literature around the over reliance on cultural approaches to examine and understand migrant health outcomes and disparities [2, 3, 5]. Central to cultural explanations is the concept of acculturation, the individual-level process through which one sheds the cultural characteristics of one’s country-of-origin and adopts those associated with the host country [2, 5]. The acculturation framework underpinned several studies included in our analysis and also features in certain policy documents [30, 34, 40, 41]. Such explanations have been critiqued as they risk identifying culture as the “problem”, as well as promoting a static definition of culture which homogenises entire populations [5]. It is argued that this reduction to and problematic treatment of culture can perpetuate racial/ethnic stereotypes and inadvertently promote “victim-blaming explanations”. An individual-level cultural change-based approach also overlooks the impact of structural factors on migrant health disparities, such as the influence of migrant policies, racialisation processes, as well as economic and social integration [5]. To Brah ([22], p. 24) such approaches reduce and present migrants as “‘culturally encapsulated’ – as if ‘culture’ was something entirely separate from lived experience”.

### Critique 2: Reduction to and of language

A consistent finding within the literature is that migrants’ experiences of the Australian health system were shaped and defined by language- and communication-related barriers. Studies overwhelmingly noted that migrants, who were unable to speak and understand English, were unable to comprehend and/or effectively communicate their needs to healthcare practitioners, as well as understand English-based education resources. Often adding that as a result, some migrants were often unaware of their diagnosis or prognosis and would even undergo procedures without fully understanding the reasons for it [42, 43]. Though interpreters did, at times, facilitate communication during a care encounter, there were issues regarding confidentiality, the accuracy of the translation, as well as their “humanity” when communicating sensitive information [41, 44]. Moreover, accessing an interpreter, in the first instance, was often an issue particularly in under-resourced or rural and regional communities; requesting an interpreter could delay access to care, prolonging waiting times for appointments [45, 46]. As a result, family members, even children, would often act as interpreters for migrants. These reported issues are despite several State and Federal government health policies and strategies advocating for the effective provision of language services and supports [33, 36, 47].

Eight studies specifically examined the impact of language- and communication-related barriers on the migrant care experience. Two of these studies compared the experiences of non-English speaking migrants with Australian-born or English-speaking individuals [48, 49]. Almost all studies categorised migrants according to specific language groups, labelling migrants as, for example, “Arabic speaking” or “Chinese speaking”. In summary, such an articulation of language and communication barriers experienced by migrant patients dominates the Australian literature and health policies. Accordingly, Australian health policies overwhelmingly stress the importance of recognising the “linguistic affiliation” of migrants and the moniker ‘CALD’ appears extensively in policy documents, in empirical research and in the mainstream healthcare discourse, and CALD communities are approached based on language, as well as culture and/or religion [33, 34, 35]. The literature however conveys a sense of “linguistic homogeneity”; that is, it seems to assume that migrants originate from distinctly bounded, homogenous societies sharing the same language [16]. The importance of recognising the heterogeneity *within* language groups has, in fact, been noted in some studies discussing interpreter services. For example, Alananzeh et al. [50] report the instance of a patient from Egypt who was unable to understand the Lebanese Arabic dialect of the translator and found it difficult to convey the “meaning of the conversation”. Similarly, Blignault et al. [51], talk about a Mandarin-speaking patient who was unable to understand the Cantonese-speaking bilingual worker that the health professional had organised for a home visit. The extensive examination of language and language-related barriers in research, and the importance accorded to language in healthcare policies is perhaps understandable given the established wisdom that language is a medium for or “proxy” for culture. In a general sense, language is the symbolic representation of a people, comprising their historical and cultural background [52, 53]. Therefore, language and culture are inextricably linked, and this could indeed explain why, within the examined literature, we identified a notable focus on how both language and culture shape the migrant care experience. We argue that such reduction of migrants en-masse to their ‘linguistic affiliation’ or broad language group is problematic as it prejudicially anticipates their English-language ability and also wrongly presents migrant populations as homogenous. In doing so, migrants’ experiences of the Australian health system are narrowly defined by the ‘inevitable’ language barriers they encounter.

### Critique 3: Homogenisation and reduction of migrants’ geographic origins

The assumption that migrants originate from geographically bounded, homogenous societies was also widely evident in the literature. Apart from categorising migrants according to broad language groups or “cultural identity”, one sees migrants’ origins being referred to in broad geographic locational terms, such as Sub-Saharan Africa or South-East Asia [17, 29, 32, 40, 41, 43, 50, 54–58], for example, compared the commonalities and differences in experiences of sexual health related help-seeking and discrimination between Sub-Saharan African and South-East and East Asian migrants. Other the other hand, Hoang et al. [29] reveal that Vietnamese, Chinese and Korean women shared similar maternity care cultural practices of confinement during pregnancy. While there are notable differences, as well as similarities in the care experiences of migrants from such broad geographies, the issue is that these studies do not recognise and engage with the vast differences *within* these groups. That is, how diversity (in terms of language, religion, and cultures) across these vast geographic regions shapes the migrant care experience. There can also be considerable diversity within large countries, such as India, which has over 22 recognised languages with only 125 million people speaking English as a first, second or third language. While the majority of the population is Hindu, there are variations between and within states. For instance, North-Eastern states have a Christian majority, whereas Sikhism is the major religion in Punjab [59]. Similarly, China has at least 55 ethnic groups (other than the Han majority) – each with their unique language, culture and often religion. Moreover, while Mandarin is recognised as the national language, dialects vary widely and can be mutually unintelligible with southern varieties of Chinese [60]. This diversity is not reflected, or engaged with, in studies which described individuals’ care experiences as that of an Asian, South-East Asian, or East Asian migrant [29, 41, 54, 57].

Though much of the literature did not appreciate the problems associated with such homogenisation and reduction, some studies did offer valuable insights into and some reflections on how such a reduction and homogenisation is problematic – both on research and policy fronts. Abdelmessih et al. [49] reveal how categorising individuals as “Arab migrants” can be problematic as cultures can significantly vary between Arabic countries, as well as different regions within the same country. Due to this, Arab migrant patients suggested that there was no need for written information on cardiovascular disease that is tailored to specific cultural needs [49]. Wamwayi et al. [39] also described how cultural differences between various ethnic African groups can, in turn, equate with different beliefs and healthcare practices. We argue that homogenising migrants and reducing them to broad geographies is problematic; doing so ignores the heterogeneity within and across countries and regions, and the impact such diversity can have on migrants’ interactions with and experiences of the Australian health system.

## Discussion

### How could migrants’ interactions with and experiences of the Australian health system be conceptualised?

Our critique of the Australian literature aimed to highlight the gaps in how migrants’ care encounters and experiences have been conceptualised. Based on our above critiques and findings and drawing on the theoretical literature, our synthesising argument about how research, policy and practice could better conceptualise and understand migrants’ experiences of the Australian health system is as follows:

*The Australian scholarly literature, and State and Federal Government health policies consistently categorise migrants according to an assumed ‘cultural identity’, linguistic affiliation, and/or geographic origin. We argue that such a reduction and homogenisation problematically, narrowly approaches migrants as ‘culturally encapsulated’, prejudicially anticipates their English-language ability and, ultimately, discounts the diversity and heterogeneity within migrant populations in Australia. This reduction and homogenisation is also clearly reflected in how health policies aimed at improving migrants’ experiences of the Australian health system are articulated and operationalised. We argue that Australian (and international) research examining migrants’ care encounters should consider the multiplicity and fluidity of migrant identities. That is, the multifaceted and shifting ways that migrants define themselves during their encounters with health systems. Engagement with this notion is necessary for also understanding how migrants’ identities are co-constructed, reinforced and contested during their interactions with health professionals and their “counter-identities”* [19–22, 24]*. We argue that these understandings have important implications for the implementation of Australian health policies and programs for enhancing health systems responsiveness to the needs and expectations of migrants.*

There is a rich body of scholarship that draws attention to the multiplicity and fluidity of the “migrant identity” which health policies and researchers do not yet explicitly engage with [19, 20, 21]. Drawing on Li and Findlay ([19], p. 375) we argue that the borderline nature of the migrant identity is not only a “transitional space between one culture and another, but as an identity whose in-betweenness is fluid”, and an explicit engagement with this fluidity is necessary to understand how migrants define themselves differently across place and time, along the various phases of their migratory process [3]. Hack-Polay et al. [21] highlights the situatedness of migrants’ identities; migrants may claim certain national or linguistic identities in particular social settings (such as within migrant circles) and other identities in different milieus. Moreover, in order to navigate the new social and cultural landscape, migrants may suspend their “native” selves, including their cultural values, beliefs, as well as language [21]. Language, in particular, can be seen as an important identity factor and, for migrants, maintaining language competency (including accents and common phrases) can be seen as imperative for social integration [21]. Therefore, the multiple, shifting and sometimes contradictory identities that migrants assume during the care encounter is overlooked by studies which simply reduce migrants to their ‘cultural’ and/or ‘linguistic’ identities. This insight into how migrants’ shifting identities, impact their expectations and experiences of care is vital for developing effective approaches to enhancing health systems responsiveness [61].

Consequently, we argue that reducing and homogenising migrant patients according to their ‘cultural identity’, language group, and/or geographic origin has led to a narrow focus, within the Australian literature and State and Federal Government health policies, on how culture and language shape migrants’ experiences of the health system. As discussed in our first critique, several studies emphasised that culture is just ‘a’ part of migrant patients’ complex life, including but not limited to, their identity [32, 37]. For recently arrived migrant women, for example, the demands of resettlement are often more pressing than their sexual and reproductive health; competing social settlement needs such as housing, employment and childcare responsibilities are prioritised over accessing care for sexual and reproductive health issues [62]. These structural factors which may also shape migrants’ interactions with and experiences of the Australian health system, are inevitably overlooked by studies that focus on the cultural and linguistic barriers migrants’ encounter [5]. Moreover, as Hack-Polay et al. [21] stress, migrants’ identities are not simply bicultural—they are far more complex and relational. Therefore, apart from evolving across space and time, the “migrant identity” is also constituted relationally during one’s interactions with others and their “counter-identities”. That is, an interactant’s multiple and shifting identities [19–22, 24]. Research suggests that during their interactions with health professionals, migrants often feel like an “intruder”, “foreigner” or “visitor”. Their expectations within the healthcare interaction, which can also be relationally, temporally and spatially defined, is often that they should just be “grateful” for being in Australia; they are thus reluctant to make their needs and expectations known to healthcare providers [32, 42, 63–67]. One’s actions, words and appearances can be “significant symbols” during an interaction; interactants constantly judge these symbols and in the process co-construct, maintain, reproduce, and sometimes contest each other’s identities [24]. Destination country language (English in Australia’s case) is one such significant symbol – but not the only one; in the study by Maneze et al. [65], individuals expressed their belief that, rather than listening to what they were saying, health professionals were judging their English-language ability based on their “Asian” appearance. In this way, one’s appearance is also a significant symbol which health professionals may subconsciously judge and respond to, and, inadvertently reinforce a sense of being ‘othered' during the care encounter [24]. It is in response to these concerns and within the frame of a provider-patient interaction that the concept (and practice) of ‘cultural safety’ was proposed, and originally operationalised in New Zealand. The practice requires health professionals to “undertake a process of reflection on his or her cultural identity” to recognise the impact that their “personal culture” has on their professional practice [68]. While cultural safety does prompt health professionals to reflect on their ‘counter’ identity and its impact on their encounters with patients from diverse backgrounds, the emphasis remains on a ‘cultural identity’ and, in this way, similarly overlooks the multiple and shifting identities of health professionals.

We argue that further research is necessary to understand how migrants’ multifaceted and shifting identities frame their care experiences, how they are also relationally constructed during the healthcare encounter, and with what consequences. Table 1 outlines an agenda for future Australian and international research seeking to examine migrant’s interactions with destination country health systems. We content that further theory-informed research is necessary to help improve the design and implementation of policies and programs directed at improving health system responsiveness to the needs and expectations of migrant communities.

**Table 1:** Agenda for future Australian and international research

Agenda for future research examining migrants’ interactions with destination country health systems	
Cultural diversity	1) Explicitly acknowledge and engage with the diversity within and across the regions and countries that individuals migrate from 2) Examine culture as it intersects with other aspects of migrants’ identities (such as migration status, socioeconomic status, sexuality, age) to influence their care experiences
Linguistic diversity	3) Take into account differences in English language ability, rather than simply categorising migrants as “non-English-speaking” 4) Explicitly acknowledge and engage with the linguistic diversity that can exist within groups hailing from apparently homogenous regions and countries (including differences in dialects)
Fluidity and multiplicity of migrants’ identities	5) Understand the shifting and multiple identities migrants assume during their care encounters and its policy implications with regards to responsiveness -related expectations and policy/practice responses 6) Consider how migrants’ identities evolve over time in the host country, as well as pre- and post-migration, and the impact on their expectations and experiences of care 7) Understand how migrants’ identities and care experiences are relationally constructed during their interactions with health professionals and their “counter-identities” and the implications for cultural competence/responsiveness

While there is growing recognition of the fluidity and multiplicity of the notion of identity, there remain some key arguments around the concept which requires further examination and research [19]. Specifically, the problem of “groupism” – the assumption that identity derives from being a member of a group [23]. Within this rubric, gender, ethnicity, and social class, are seen as homogenous and a given, rather than social processes or relations. The assumptive categories reduce individuals as narrowly “belonging” to these groups which share only certain commonalities and, as a result, differences within groups – often vast and very important—are ignored. Drawing on Anthias [23], we contend that this reduction is a result of an uncritical resorting to – by researchers and policy-makers alike—commonly held, often lay notions and assumptions that migrants belong to certain ‘ethnic groups’ and represent certain ‘cultural’ predispositions. These assumptions are complicated by the again ‘common’ conflation between identity and culture despite clear and nuanced scholarly insight that in social interactions individuals embody and strategically mobilise not just their ethnic and cultural identities, but also other identity constructs like diverse gender identities, sexual orientation and class [23]. Moreover, an intersectionality-informed approach recognises that such social structures and relations *intersect* to shape power relations that operate in various social interactions (such as the patient-practitioner interaction). Differences in power, structured along these intersections, produce inequities that shape individuals’ experiences. We argue that this consideration is overlooked by a narrow focus on peoples’ ethnic and cultural identities [23, 25, 67]. We argue that the reduction and homogenisation – as reflected in the simplistic categorisation of migrants according to cultural and/or linguistic groups – while somehow understandable, potentially subverts and frustrates both, our understanding of migrants’ care encounters and experiences, and the ability of destination country health system to be responsive to the needs and expectations of all those they serve.

## Limitations

Depraetere et al. [10] highlight that there can be substantial inter-study variability in the processes and reporting standards of CIS reviews. We recognised this limitation and have tried to thoroughly report our literature selection, and quality assessment processes. Templier and Paré [69] argue that transparent reporting of key procedures is essential for the trustworthiness and overall quality of any review. Thus, we have been very transparent about our analytical approach i.e. the use of an abductive analytical approach to the process of synthesis, recognising that this approach is different to the inductive meta-ethnography technique used in most CIS. We also clearly outlined the method of critical appraisal which involved prioritising the relevance or theoretical contribution of studies over methodological quality. We recognise that a potential limitation of such a prioritisation is the inclusion of methodologically weak papers—and that it may have an impact on the inferences (critiques) and ultimately the final synthesising argument. However, during the process of critical appraisal of the literature we also carefully examined the methodological quality of each paper, excluding those which were clearly of poor quality.

## Conclusion

More than ever, people are migrating across countries, and destination country health systems are recognising the complexities of migrants’ journeys and lives. Over the last two decades, major destination countries, like Australia, have instituted many policies and are implementing various initiatives directed at improving the responsiveness of their health systems to the needs and expectations of migrant communities. In this critical interpretive synthesis, we offer a theory-informed critique of Australia’s policies and their implementation. We contend that both research and policy should explicitly recognise and engage with the multifaceted and shifting ways that migrants define themselves, generally, and during their encounters with destination country health systems. Engagement with this notion is necessary for also understanding how aspects of migrants’ identities are dynamically co-constructed during their interactions with the health system. These nuanced insights can help to improve the design and implementation of policies and programs directed at improving the responsiveness of Australia’s health system to the needs and expectations of migrant communities specifically, and destination countries broadly.

## Supplementary Information

**Additional file 1.** Preferred Reporting Items for Systematic Reviews and Meta-Analyses (PRISMA) Checklist.

**Additional file 2.** Data Extraction Tables.

**Additional file 3.** Development of critiques and synthesising argument.

## Data Availability

Data sharing is not applicable to this article as no datasets were generated or analysed during the current study.
