# Supplementary material for: A critical interpretive synthesis of migrants’ experiences of the Australian health system
Source: Int J Equity Health. 2023 Jan 9;22:7. doi: 10.1186/s12939-022-01821-2 (PMC9827657; doi:10.1186/s12939-022-01821-2)
Supplement: Supplementary file 1 — Additional file 1. Preferred Reporting Items for Systematic Reviews and Meta-Analyses (PRISMA) Checklist. [file 12939_2022_1821_MOESM1_ESM.docx]

| **PRISMA Checklist** | | | |
| --- | --- | --- | --- |
| **Section** | **Item** | **Checklist item** | **Page number it is reported** |
| **TITLE** | | | |
| Title | 1 | Identify the report as a systematic review. |  |
| **ABSTRACT** | | | |
| Abstract | 2 | Provide a structured summary that includes: background (objectives), methods (eligibility criteria, information sources,), results (included studies and synthesis), discussion (limitations and interpretation), funding and registration. |  |
| **INTRODUCTION** | | | |
| Rationale | 3 | Describe the rationale for the review in the context of existing knowledge. | 4 |
| Objectives | 4 | Provide an explicit statement of the objective(s) or question(s) the review addresses. | 4 |
| **METHODS** | | | |
| Eligibility criteria | 5 | Specify the inclusion and exclusion criteria for the review and how studies were grouped for the syntheses. | 6 |
| Information sources | 6 | Specify all databases, registers, websites, organisations, reference lists and other sources searched or consulted to identify studies. Specify the date when each source was last searched or consulted. | 5 |
| Search strategy | 7 | Present the full search strategies for all databases, registers and websites, including any filters and limits used. | 5 |
| Selection process | 8 | Specify the methods used to decide whether a study met the inclusion criteria of the review, including how many reviewers screened each record and each report retrieved, whether they worked independently, and if applicable, details of automation tools used in the process. | 5-6 |
| Data collection process | 9 | Specify the methods used to collect data from reports, and if applicable, details of automation tools used in the process. | 7-8 |
| Data items | 10 | List and define all outcomes for which data were sought. List and define all other variables for which data were sought (e.g. participant and intervention characteristics, funding sources). | N/A |
| Critical appraisal^§^ | 11 | Quality of studies critically appraised by prioritising likely relevance of the paper over methodological quality. | 7 |
| Effect measures^‡^ | 12 | Specify for each outcome the effect measure(s) (e.g. risk ratio, mean difference) used in the synthesis or presentation of results. | N/A |
| Synthesis methods | 13 | Describe the processes used to decide which studies were eligible for each synthesis and the methods used to synthesise results. | 7-8 |
| Reporting bias assessment ^§^ | 14 | Describe any methods used to assess risk of bias due to missing results in a synthesis. | N/A |
| Certainty assessment^§^ | 15 | Describe any methods used to assess certainty (or confidence) in the body of evidence for an outcome. | N/A |
| **RESULTS** | | | |
| Study selection | 16 | Describe the results of the search and selection process, from the number of records identified in the search to the number of studies included in the review, ideally using a flow diagram. Cite studies that might appear to meet the inclusion criteria, but which were excluded, and explain why they were excluded. | 9 |
| Study characteristics | 17 | Cite each included study and present its characteristics. | 9 |
| Critical appraisal studies | 18 | Present assessments of the critical appraisal for included studies. | 7 |
| Results of individual studies^‡^ | 19 | For all outcomes, present, for each study: (a) summary statistics for each group (where appropriate) and (b) an effect estimate and its precision (e.g. confidence/credible interval), ideally using structured tables or plots. | N/A |
| Results of synthesis^‡^ | 20 | Summarise and present results of synthesis conducted in the form of a synthesising argument. | 9-15 |
| Reporting biases^§^ | 21 | Present assessments of risk of bias due to missing results. | N/A |
| Certainty of evidence^§^ | 22 | Present assessments of certainty (or confidence) in the body of evidence for each outcome assessed. | N/A |
| **DISCUSSION** | | | |
| Discussion | 23 | Provide a general interpretation of the results in the context of the other evidence. Discuss the limitations of the evidence included in the review and the review processes used. Discuss implications for practice, policy and future research. | 14-18 |
| **OTHER INFORMATION** | | | |
| Registration and protocol | 24 | Registration information of review and/or protocol | N/A |
| Support | 25 | Sources of financial or non-financial support for the review. | 22 |
| Competing interests | 26 | Declare any competing interests of review authors. | 22 |
| Availability of data | 27 | Report which of the following are publicly available and where they can be found: template data collection forms; data extracted from included studies; data used for all analyses; analytic code; any other materials used in the review. | 22 |

**Additional File 1: Preferred Reporting Items for Systematic Reviews and Meta-Analyses (PRISMA) Checklist**

§The term “critical appraisal” is used instead of “risk of bias” in items 11 and 18 as Dixon-Woods et al. (2006) propose an alternative approach to quality assessment than which is used in conventional systematic reviews. This approach prioritises the likely relevance of a paper over methodological quality. Items 14, 15, 21 and 22 are therefore not applicable.

‡ As opposed to conventional systematic reviews, the aim of a CIS is the development of a synthesising argument: a critically informed integration of evidence from across the studies in the review. As a result, items 12 and 19 are not applicable.

*Adapted From:*  Page MJ, McKenzie JE, Bossuyt PM, Boutron I, Hoffmann TC, Mulrow CD, et al. The PRISMA 2020 statement: an updated guideline for reporting systematic reviews. BMJ 2021;372: n71. doi: 10.1136/bmj. n7
