# Supplementary material for: A critical interpretive synthesis of migrants’ experiences of the Australian health system
Source: Int J Equity Health. 2023 Jan 9;22:7. doi: 10.1186/s12939-022-01821-2 (PMC9827657; doi:10.1186/s12939-022-01821-2)
Supplement: Supplementary file 2 — Additional file 2. Data Extraction Tables. [file 12939_2022_1821_MOESM2_ESM.docx]

**Additional File 2: Data Extraction Tables**

| Empirical literature | | | | | | |
| --- | --- | --- | --- | --- | --- | --- |
| Record | **Methodology** | **Perspective** | **Aims** | **Type of health service/issue examined** | **Migrant population(s) examined** | **Theoretical framework used in analysis** |
| Abdelmessih et al. (2019) | Qualitative | Patient | To explore the health care challenges and needs of Arabic-speaking immigrants with CVD compared with English-speaking patients with CVD. | Cardiovascular disease | Arabic-speaking migrants compared to Caucasian, English-speaking individuals | N/A |
| Agu et al. (2016) | Qualitative | Patients and key informants | To explore the barriers and enablers to accessing sexual health services for sub-Saharan African, Southeast Asian, and East Asian migrants. | SRH services | Migrants from Sub-Saharan Africa and Southeast Asia | N/A |
| Alananzeh et al. (2018) | Qualitative | Patient | To understand Arab cancer survivors’ experiences with healthcare interpreters. | Cancer care (specifically interpreter services) | Arab migrant patients | N/A |
| Alananzeh et al. (2019) | Mixed-methods | Patient or carer | To explore the information needs and preferences for information delivery among Arab cancer survivors and their family caregivers in Australia (Arab migrants) and in Jordan (native Arab). | Cancer care | Arab migrants (compared with 'native Arabs' in Jordan) | N/A |
| Alzayer et al. (2018) | Qualitative | Patient or carer | To explore the asthma management experiences and perspectives of Arabic-speaking people with asthma, who have low English proficiency. | Asthma management | Arabic-speaking migrants | N/A |
| Alzubaidi et al. (2015) | Qualitative | Patient | To explore the decision-making process to access and use of healthcare services and identify associated barriers in Arabic-speaking and English-speaking Caucasian patients with Type 2 Diabetes. | Type 2 Diabetes and health services in general | Arabic-speaking migrants born in the Middle East compared to Caucasian, English-speaking individuals | N/A |
| Alzubaidi, McNamara and Browning (2017) | Qualitative | Patient | To explore a new model for diabetes self-management support for Arabic-speaking migrants. | Diabetes self-management | Arabic-speaking migrants | N/A |
| Anaman-Torgbor, King and Correa-Velez (2017) | Qualitative | Patient | To understand barriers to, and facilitators of, cervical screening practices among African women from refugee and non-refugee backgrounds in Brisbane. | Cervical cancer screening | African-born women | N/A |
| Antoniades, Mazza and Brijnath (2018) | Qualitative | Patient | To examine the interplay between culture, social networks and health seeking behaviours across two culturally distinct communities; Sri Lankans residing in Australia and Anglo-Australians. | Mental health | Sri Lankan migrants compared to Anglo-Australians | N/A |
| Benza and Liamputtong (2017) | Qualitative | Patient | To understand how Zimbabwean women perceive motherhood, their perceived roles of motherhood and their experiences of motherhood. | Maternity care | Zimbabwean women | Moral career of motherhood theory |
| Blignault et al. (2008) | Qualitative, but survey included during the interviews | Healthcare provider, patient, carer and community | To examine what factors influence the low utilisation of mental health services for the China-born migrants now living in Australia. | Mental health | Chinese-born migrant women | N/A |
| Botfield, Newman and Zwi (2017) | Qualitative | Healthcare provider, policymakers, academics and community advocates | To understand professionals’ views on engaging young migrants in Australian SRH services. | SRH services | Young migrants and refugees (country-of-origin not specified) | Cultural competence framework |
| Botfield, Newman and Zwi (2018) | Qualitative | Patient | To explore the complexities and opportunities for engaging young migrants in sexual and reproductive health information and care. | SRH | Young migrants and refugees (country-of-origin not specified) | N/A |
| Botfield et al. (2018) | Qualitative | Patient and expert informants | To explore the complexities and opportunities for engaging young migrants in sexual and reproductive health information and care. | SRH services | Young migrants and refugees (country-of-origin not specified) | N/A |
| Botfield et al. (2020) | Qualitative | Patient | To present a secondary analysis of qualitative data related to unintended pregnancy and abortion in a study exploring the views and experiences of young people from migrant and refugee backgrounds. | SRH services | Young migrants and refugees (country-of-origin not specified) | N/A |
| Boughtwood et al. (2011) | Qualitative | Carer | To provide an examination of the experiences and perceptions of family carers of the people living with dementia from the four Australian CALD communities. | Dementia | Italian-, Chinese-, Spanish- and Arabic-speaking migrants | N/A |
| Boughtwood et al. (2012) | Qualitative | Carers and health professionals | To explore how CALD communities access information about dementia, what are the main access issues and how can information provision be improved. | Dementia | CALD family caregivers (Arabic-, Chinese-, Italian- and Spanish-speaking), bilingual and bicultural workers, bilingual general practitioners and geriatricians | N/A |
| Broom et al. (2019) | Qualitative | Healthcare provider | To explore how professionals in cancer care experience their encounters with migrant cancer patients. In particular, how they work with cultural diversity in their everyday practice, and the personal, interpersonal and institutional dimensions therein. | Cancer care | Not specified | Various sociological cultural perspectives |
| Broom et al. (2020) | Qualitative | Patients and healthcare professionals | To explore the relational and coproduced nature of difference in the accounts of professionals and patients from migrant backgrounds, in the context of cancer care. | Cancer care | Migrant women from various countries-of-origin | Relationality |
| Butow et al. (2010) | Qualitative | Patient or carer | To identify healthcare-related communication issues for migrants who develop cancer and factors associated with these challenges. | Cancer care | Arabic-, Chinese- or Greek-speaking migrants | Acculturation scale |
| Carolan and Cassar (2008) | Qualitative | Patient | To examine the experiences of African women receiving pregnancy care in Melbourne, Australia.  In particular, it aimed to explore African women’s experiences of Australian antenatal care from an ‘emic’ perspective. | Antenatal care | African-born migrant and refugee women | N/A |
| Carolan-Olah and Cassar (2018) | Qualitative | Patient | To evaluate elderly Italian migrants’ experience of diabetes care and the factors that may contribute to local health service use. | Type 2 diabetes | Older Italian migrants | N/A |
| Chan and Ritchie (2011) | Qualitative | Patient | To further explore what motivated Chinese migrants to seek help for emotional distress and their reasons for their chosen behaviour. | Mental health | Chinese migrants | N/A |
| Choi, Walker and Palermo (2018) | Qualitative | Healthcare providers and patients | To understand the experiences of Chinese migrants living with type 2 diabetes in Australia, and explore their culturally specific diabetes management habits, needs and expectations in the Australian context. | Diabetes self-management | Chinese migrants | N/A |
| Cross and Bloomer (2010) | Qualitative | Healthcare professionals and community mental health workers | To explore the issues confronted by mental health clinicians when communicating with and providing a mental health service for people from CALD communities. | Mental health | CALD community in general | N/A |
| Davidson et al. (2011) | Qualitative | Patients, community members (focus groups) and Chinese-born health professionals | To describe the experiences of Chinese Australians with heart disease following a discharge from hospital for an acute cardiac event. Specifically:   1. To identify patterns and cultural differences of Chinese Australians following discharge from hospital 2. Illustrate the illness/health seeking behaviours and health beliefs of Chinese Australians. | Cardiovascular disease | “Chinese Australians” | N/A |
| Dolan et al. (2020) | Qualitative | Healthcare professionals | To explore healthcare professionals’ experiences with providing contraceptive services to Chinese migrant women. Specially, to investigate their perceptions of Chinese migrant women’s information, communication and support needs, as well their own needs in supporting Chinese women’s decision-making. | Contraception | Chinese migrant women | Ottawa Decision Support framework |
| Garg et al. (2017) | Qualitative | Parents | To understand the factors influencing CALD parents’ access of primary health care services for developmental surveillance and anticipatory guidance for their children. | Child health surveillance service | “CALD community” | An ecological model |
| Gilbert, Antoniades and Brijnath (2019) | Qualitative | Patient | To examine, through a framework of trust, how Indian migrants negotiate the Australian healthcare system, as well as their use of transnational treatment options. | Healthcare services in general | Indian migrants | Theorisations of trust |
| Gray et al. (2019) | Qualitative | Patient | To gain a better understanding of the barriers to HIV testing for people born in Sub-Saharan Africa and Southeast/Northeast Asia. | HIV | Migrants from Sub-Saharan Africa and Southeast/Northeast Asia | N/A |
| Hannah and Le (2012) | Qualitative | Patient | To examine the factors affecting access to healthcare services for intermarried Filipino women in rural Tasmania. | Health services in general | Filipino migrant women | N/A |
| Harrison et al. (2019) | Qualitative | Bilingual health workers and patients | To capture the perceptions of CALD consumers regarding the barriers to, and enablers of, engagement in health care through native language focus group inquiry. | Health services in general but with a focus on interpreter services | “CALD community” | N/A |
| Henderson and Kendall (2011) | Qualitative | “Community navigators” | To explore the ‘lived experience’ of the “community navigators” who | Healthcare services in general | CALD community (Specifically Burmese, Afghan, Sudanese and Pacific Islander migrants) | N/A |
| Hoang, Le and Kilpatrick (2009) | Qualitative | Patient | To investigate the childbirth experiences of Asian migrants living in rural Tasmania. | Maternity care | Asian migrant women | N/A |
| Hoban and Liamputtong (2013) | Qualitative | Patient | To explore the postpartum experiences of Cambodian born migrant women who gave birth for the ﬁrst time in Victoria, Australia. | Pregnancy and the postnatal period | Cambodian migrant women | N/A |
| Hughson et al. (2018) | Qualitative | Maternity care staff | 1. To identify the barriers to providing pregnancy-related information to CALD women in the Australian setting, with a particular focus on health literacy issues. 2. To examines health professionals’ perspectives towards using electronic maternity health information as an enabler to providing information to a CALD patient population. | Maternity care | “CALD community” | Framework of cultural competence education interventions for health professionals and a health literacy framework |
| Ilami and Winter (2020) | Qualitative | Patient | To investigate the perceptions and experiences (barriers and enablers) of Iranian migrants in accessing (public and private) healthcare services and information for their sexual and reproductive health needs. | SRH services | Iranian migrants | N/A |
| Javanparast, Naqvi and Mwanri (2020) | Qualitative | Healthcare provider | To explore the factors that influence access to and utilisation of health services among CALD populations in regional South Australia. | Regional health services | “CALD community” | “Access to health service” theoretical framework developed by Levesque and colleagues |
| Jin et al. (2020) | Qualitative | Patient and/or family member | To investigate the multilevel and interactive elements of individual, family, institutional, community, and policy factors that influence engagement with coronary heart disease primary and secondary prevention among Chinese migrants and their family carers | Coronary heart disease | Chinese migrants | Socioecological framework |
| Johnstone et al. (2016) | Qualitative | Health professionals (nurses) | To explore and describe the strategies nurses use to facilitate engagement with families of older immigrant NESB patients hospitalised for end-of-life care. | End-of-life care | Older non-English-speaking background (NESB) immigrants | N/A |
| Johnstone et al. (2016) | Qualitative | Health professionals (nurses) | To explore and describe nursing roles and strategies in end-of-life decision making and their possible link to enabling a good death for elderly immigrants. | End-of-life care | Older non-English-speaking background (NESB) immigrants | N/A |
| Johnstone et al. (2018) | Qualitative | Health professionals (nurses) | To explore and describe the specific processes that nurses use to foster trust and overcome possible cultural mistrust when caring for older immigrants of non-English speaking backgrounds hospitalised for end-of-life care. | End-of-life care | Older non-English-speaking background (NESB) immigrants | N/A |
| Jowsey, Gillespie and Aspin (2010) | Qualitative | Patient or carer | To examine how non English-speaking migrants’ experiences of diabetes management compare with those of people born in Australia. In particular, how their experiences influence their ability to self-manage their condition. | Diabetes self-management | Non English-speaking migrants compared to Australian-born | N/A |
| Kokanovic and Manderson (2007) | Qualitative | Patient | To explore the perceptions of Australian immigrants on their interactions with doctors regarding the diagnosis, treatment, and management of type 2 diabetes mellitus. | Type 2 Diabetes | Migrants from various countries-of-origin | N/A |
| Komaric, Bedford and van Driel (2012) | Qualitative | Patients and healthcare professionals | To describe the challenges and barriers people from CALD communities face regarding treating and preventing a chronic disease, as well access to health services. | Chronic illness | “CALD community” (Arabic-speaking background, or born in Sudan, China, Vietnam or Tonga) | N/A |
| Körner (2007) | Qualitative | Patient | To describe the interrelationships between migration and resettlement, the Australian immigration system and living with HIV. | HIV | Migrants from various countries-of-origin | N/A |
| Lang et al. (2020) | Qualitative | Patient | To explore pregnancy planning, preconception lifestyles, awareness, experiences and healthcare needs of migrant women in Australia. | Preconception health | Migrant women from various countries-of-origin | N/A |
| Levesque et al. (2020) | Qualitative | Patient | To explore the experience of breast cancer and coping behaviours utilised by Chinese migrant women. | Breast cancer | Chinese migrant women | N/A |
| Lim et al. (2019) | Qualitative | Patient | To describe the challenges and unmet information needs of the Chinese community in Australia affected by cancer, and outline the barriers that prevent optimal cancer care for this community. | Cancer care | Chinese migrants | N/A |
| Maharaj and Bandyopadhyay (2013) | Qualitative | Patient | To investigate the influence of culture and identity on the immigrant experience of early motherhood (specifically related to breastfeeding) among ethnic Indian. | Breastfeeding and maternity care | Indian migrant women from Indian, Fiji, South Africa and the United Kingdom | Theories on acculturation and identity |
| Maneze at al. (2015) | Mixed-methods | Patient | To explore Filipino migrants’ perceptions of facilitators and barriers to maintaining health in Australia. | Healthcare services in general | Filipino migrants | Ecological framework |
| Maneze et al. (2016) | Qualitative | Patient | To explore the experiences of Filipino migrants with chronic diseases when communicating with healthcare professionals during clinical encounters. | Chronic illness | Filipino migrants | N/A |
| Maneze et al. (2018) | Qualitative | Patient | To examine the experiences of Filipino-Australian migrants with chronic disease, their management of chronic conditions in their adopted country, and how these experiences may influence health promotion and health-seeking behaviours. | Chronic illness | Filipino migrants | N/A |
| Marshall et al. (2020) | Qualitative | Patients and health professionals | To gain further understanding of formal and informal infant feeding supports among Arabic and Chinese speaking migrant mothers, through the perspectives of mothers and health professionals. | Breastfeeding | Arabic- and Chinese- speaking migrant women and health professionals working with Arabic- and Chinese-speaking patients | N/A |
| McCann et al. (2016) | Qualitative | Patients, parents and key community leaders | To identify the help-seeking barriers and facilitators for anxiety, depression and alcohol and drug use problems in young recent migrants from sub-Saharan Africa. | Mental health and/or substance abuse | Sub-Saharan African migrants (also included under the term ‘migrant’ were those who entered through the Humanitarian and Refugee program) | N/A |
| Mengesha et al. (2017) | Qualitative | Healthcare provider | To examine HCP’s perceptions of the challenges and barriers influencing refugee and migrant women’s access and utilisation of SRH. | SRH services | Migrants and refugees from various countries-of-origin | Socioecological framework |
| Mengesha et al. (2018) | Mixed-methods | Healthcare provider | 1. Assess the perceived knowledge and conﬁdence of HCPs in their ability to work with refugee and migrant women seeking SRH care 2. Examine HCP’s training experiences and needs with respect to the provision of SRH care to refugee and migrant women in Australia. | SRH services | Migrants and refugees from various countries-of-origin | Socioecological framework |
| Minas et al. (2013) | Review | N/A | 1. To examine what is known about the mental health of immigrant and refugee communities in Australia 2. Whether Australian mental health research pays adequate attention to the CALD population 3. Whether national mental health data collections support evidence-informed mental health policy and practice in multicultural Australia | Mental health | Migrants in general | N/A |
| Mohale, Sweet and Graham (2017) | Qualitative | Patient | To examine the experiences of Sub-Saharan African women in relation to maternity care services in their home countries and in Australia. | Maternity care | Sub-Saharan African migrant women (women who gave birth both in Sub-Saharan Africa and Australia) | N/A |
| Mohan Wilkes and Jackson (2006) | Qualitative | Carer | To explore the experiences of the family members of Asian Indians with coronary heart disease. | Coronary heart disease | Indian migrants | N/A |
| Mollah et al. (2018) | Qualitative | Mental health practitioners | To explore what frontline practitioners consider to be culturally competent care and what helps or hinders them in delivering such care. | Mental health | Migrant women from various countries-of-origin | N/A |
| O’Callaghan et al. (2016) | Qualitative | Patient | To understand the conceptualisation of the term ‘survivor’ and survivorship issues and information needs amongst Chinese- and Greek-speaking cancer survivors. | Cancer care | Chinese- and Greek-speaking migrants | N/A |
| Ogunsiji et al. (2013) | Qualitative | Patient | To explore West African migrant women’s knowledge, attitude and usage of cancer screening in Australia. | Breast/cervical cancer screening | West-African migrant women (Ghanaian and Nigerian) | N/A |
| Owens, Dandy and Hancock (2016) | Qualitative | Patient | To gain an understanding of the lived experiences of migrant women of CALD backgrounds who have used community-based antenatal services. | Community-based antenatal care | Migrant and refugee women from various countries-of-origin | Models of care conceptual framework |
| Rao, Dahlen and Razee (2020) | Qualitative | Patient | To explore the experiences of motherhood and postpartum support of Indian migrant mothers. | Maternity care (specifically postnatal care) | Indian migrant women | N/A |
| Raymundo, Smith-Merry and McNab (2021) | Qualitative | Patient | To explore young migrants’ experiences of accessing health services and identify barriers and facilitators to health service utilisation. | Healthcare services in general | Young migrants (including second-generation migrants) | N/A |
| Rehayem et al. (2020) | Mixed-methods | Patient | To explore the experiences, knowledge and inﬂuences relating to infant feeding in Arabic-speaking women in Australia. | Breastfeeding | Arab migrant women (Christian and Muslim faiths) | N/A |
| Renzhao (2008) | Qualitative | Managers and direct service providers from various community health agencies | To document how service providers identify and develop services to meet the needs of CALD communities. | Culturally competent healthcare provision | CALD community in general | Cultural competence |
| Renzaho and Oldroyd (2014) | Qualitative | Patient | To explore the views and perceptions of migrant women on sociocultural barriers and health needs during pregnancy and in the postnatal period. | Maternity care | Afghani, African, Chinese and Middle Eastern migrant women | Social identity theory and acculturation |
| Robotin et al. (2017) | Qualitative – literature review and development of multimedia resource | Community consultation | To build a better understanding of the unmet information needs experienced by Vietnamese and Chinese migrants aﬀected by liver cancer. | Cancer care (Hepatitis B) | Vietnamese and Chinese migrants | N/A |
| Rodriguez (2013) | Qualitative | Patients and key informants | To examine Polynesian migrants’ perceptions of health and illness, and their experience of health services in New South Wales. | Healthcare services in general | Polynesian migrants | Kaupapa Maori and cultural safety frameworks |
| Rogers and Earnest (2014) | Qualitative | Patient | To examine the inter- generational experiences and knowledge of reproductive health and contraception among Sudanese and Eritrean migrant and refugee mothers and daughters. | SRH services | Sudanese and Eritrean migrants | Psychosocial framework |
| Saleh et al. (2012) | Qualitative | Patients or community members | To explore with Arabic-Australian patients and their communities, the cultural context of cancer by examining their beliefs about causes, as well as modes of communication about cancer with family, friends and the community. | Cancer care | Arab migrants | N/A |
| Shafiei, Small and McLachlan (2015) | Qualitative | Patient | To investigate immigrant Afghan women's emotional wellbeing and experiences of postnatal depression after childbirth and their use of health services. | Maternity care | Afghani migrant women | N/A |
| Shafiei (2015) | Mixed-methods | Patient | To explore immigrant Afghan women’s views and experiences of maternity care in Melbourne, Australia. | Maternity care | Afghani migrant women | N/A |
| Shanmugasundaram and O'Connor (2009) | Qualitative | Carer | To explore the issues related to accessing palliative care services for Indian migrants, identify the effectiveness of palliative care in supporting the patient and family, and to make recommendations for improving care for the family members of terminally ill Indian migrants. | Palliative care | Indian migrants | N/A |
| Shanmugasundaram (2015) | Qualitative | Carer | To highlight the needs of the family caregivers of Indian patients receiving palliative care services in Australia. | Palliative care | Indian migrants | N/A |
| Shaw et al. (2013) | Qualitative | Patient or carer | To explore migrant cancer patients’ experiences of cancer care coordination during treatment. | Telephone-based supportive cancer care intervention | Chinese- and Arabic- speaking migrants | N/A |
| Shaw, Zou and Butow (2015) | Qualitative | Patient or carer | To explore the factors that inﬂuence the cancer treatment decision-making experiences of ﬁrst generation migrants with cancer from Arabic, Chinese, or Greek backgrounds. | Cancer care | Arabic, Chinese or Greek first-generation migrants | N/A |
| Shaw et al. (2016) | Qualitative | Patient or carer | 1. To identify cultural sensitivities that are important to the acceptability of the telephone-based intervention 2. To identify cultural barriers and facilitators to intervention participation. | Cancer care | Chinese-, Arabic- and Macedonian-speaking migrants | N/A |
| Sneesby et al. (2011) | Qualitative | Community members | To obtain information to support palliative care healthcare workers to meet the needs of the Sudanese population in death, dying and bereavement. | Palliative care | South Sudanese | N/A |
| Stanzel et al. (2020) | Qualitative | Patient | 1. To explore how Vietnamese-born women who migrated to Australia in adulthood manage the menopausal transition and their postmenopausal health 2. How women access, understand, evaluate and use menopause-related health resources 3. To examine their experiences with menopause-related health care. | Menopause | Peri- or post-menopausal Vietnamese migrant women | The Integrated Model of Health Literacy |
| Stanzel, Hammarberg and Fisher (2021) | Qualitative | Patient | 1. To describe how women, who were born in the Horn of Africa nations and migrated to Australia in adulthood, experience and manage their menopausal transition. 2. How personal, situational, societal and environmental factors influence their health literacy skills, 3. To explore their experiences and satisfaction with menopause-related health care. | Menopause | Migrant women from Horn of Africa nations | N/A |
| Suwankhong and Liamputtong (2018) | Qualitative | Patient | To explore Thai migrant women’s perceived susceptibility to breast cancer and barriers to early breast cancer screening. | Breast cancer screening | Thai migrant women | Health belief model |
| Terry, Ali and Le (2011) | Qualitative | Patient | To identify the acculturation process by which Asian migrants are enabled to use the health system. | Healthcare services in general | Asian migrants | Acculturation but in terms of “health system acculturation” |
| Truong et al. (2017) | Qualitative | Healthcare provider | To explore healthcare provider perceptions of cultural competence in community health service provision in terms of:   1. Their perceptions of the significance of client cultural background 2. How they understand the concept of cultural competence, 3. Other factors related to cultural competence. | Community health services and cultural competence | Not specified | Cultural competence framework |
| Turkmani, Homer and Dawson (2020) | Qualitative | Patient | To identify approaches to achieve culturally safe and high-quality woman-centred care for migrant women who have been personally aﬀected by FGM. | Maternity care (specifically for women affected by FGM/C) | Migrant and refugee women affected by FGM | N/A |
| Varol et al. (2017) | Review | N/A | Review the literature on research on FGM/C in Australia, which focuses on health system response to women and girls with FGM/C. | Healthcare services in general | Migrant women and girls who have experienced FGM/C | N/A |
| Vatcharavongvan et al. (2014) | Qualitative | Patient | To explore the health needs of Thai migrants in Brisbane, Australia, and how health, familial and social problems are addressed by this local Thai community. | Healthcare services in general | Thai migrants | N/A |
| Wah et al. (2019) | Qualitative | Patient | To explore the understanding and self-management experiences of gestational diabetes management among Chinese migrants. | Maternity care, specifically, gestational diabetes self-management | Chinese migrant women | N/A |
| Wamwayi, Cope and Murray (2019) | Qualitative | Healthcare provider | To establish a baseline of knowledge regarding the provision of care for African immigrant patients in a mental health setting. | Mental health | African refugees and migrants | N/A |
| Wand, Pourmand and Draper (2020) | Editorial | N/A | To examine what we know about using interpreters with culturally and linguistically diverse older adults. | Interpreter services | Older non-English-speaking background (NESB) migrants | N/A |
| Wilson and Hughes (2017) | Review | N/A | To examine how to preventing lifestyle- related disease among recently arrived immigrants by partnering with English language providers to improve cancer literacy. | Cancer care and health literacy | Migrants in general | N/A |
| Wohler and Dantas (2017) | Review | N/A | To provide an overview of the reported barriers accessing mental healthcare by CALD women, drawing on findings from a systematic review assessing the mental health of CALD women in Australia. | Mental health | CALD women | N/A |
| Youssef and Deane (2006) | Mixed-methods | “Key informant” – Religious leaders and bilingual health workers | To investigate the major issues that facilitate or hinder effective utilisation of mental health services by Arabic-speaking people in Sydney. | Mental health | Arabic-speaking migrants | N/A |
| Zulfiqar et al. (2017) | Qualitative | Patient | To explore the barriers and facilitators to long-term healthy lifestyle recommendations among Australian-born and overseas-born-women with respect to gestational diabetes. | Postnatal care, specifically, post gestational diabetes | Migrant women from a range of backgrounds, compared to Australian-born women. | N/A |

| Policies | | | | | |
| --- | --- | --- | --- | --- | --- |
| Record | **Year** | **Health issue/service** | **Targeted population group** | **Proposed strategy/initiative** | **Conceptualisation of cultural responsiveness/competence (if relevant)** |
| Cultural responsiveness framework: Guidelines for Victorian health services, Victorian Department of Health | 2009 | Health services in general. In response to a lack of consensus on the definition of cultural competence. | CALD community | A cultural responsiveness framework that targets four domains:  Organisational effectiveness, risk management, consumer participation and effective workforce. | Cultural responsiveness–   - “Refers to health care services that are *respectful of,* and *relevant to,* the health beliefs, health practices, culture and linguistic needs of diverse consumer/patient populations and communities”. - More specifically, “communities whose members identify as having particular cultural or linguistic affiliations by virtue of their place of birth, ancestry or ethnic origin, religion, preferred language or language spoken at home.” |
| Culturally and Linguistically Diverse Children and their families – Implications for paediatric and child development services in Queensland, Queensland Health | 2019 | Paediatric and child development services. In response to the multicultural diversity increasing throughout Queensland, due to increased migration and refugee intake. | CALD community | To build a common understanding around cultural considerations for health practitioners in child development and outline a commitment to a culturally capable workforce. | - Culture is seen as a “dynamic yet stable set of beliefs and attitudes shared by a group of people”, that influences how we experience and manage health and illness. - “Culturally responsive practice” – a set of “congruent behaviours, attitudes and policies that come together in a system or agency or among professionals’ that enable effective work in cross- cultural situations” - Linked with family-centred practice as culture profoundly shaped both human development and family structures. |
| Delivering for diversity: Cultural diversity plan 2016–2019, Department of Health and Human Services | 2016 | Embedding of the cultural diversity plan within the department’s services, programs and policies. In response to obligations under the *Multicultural Victoria Act 2011*. | CALD community | Responding to cultural diversity through four outcome areas:   1. Equitable and responsive services and programs 2. A culturally responsive workforce 3. Partnership with diverse communities 4. Effective and evidence-based approaches | - Cultural responsiveness can be facilitated through overcoming barriers to access, using a range of communication strategies, the effective delivery of language services and implementing cultural responsiveness measures. - A culturally responsive health workforce also facilitated through building capabilities of cultural responsiveness and competence and promoting CALD recruitment. |
| Exploratory Analysis of Barriers to Palliative Care:  Issues Report on People from Culturally and Linguistically Diverse Backgrounds, Australian Government Department of Health | 2019 | To conduct an exploratory analysis of barriers to accessing quality palliative care for people from under-served populations or people with complex needs. The report describes key barriers and promising approaches for improving CALD populations’ access to and experience of palliative care in Australia. | CALD community | Seven facilitators of quality palliative care were identified underpinned by four enablers. Palliative Care Victoria also developed the *Culturally Responsive Palliative Care Strategy*. | The report recognises that CALD populations are diverse, with differences in language, cultural norms and religious beliefs. However, “culture plays a critical role in how patients, families, and healthcare providers view the end-of-life”. This underpins the *Culturally Responsive Palliative Care Strategy,* as well as recommendations of improving cultural competence within services. |
| National Standards for Mental Health Services, Australian Government | 2010 | Mental health services in general. In response to a revision of the National Standards for Mental Health introduced in 2006 | Australian population, in general | Proposal of 10 National Standards, with Standard 4 (Diversity responsiveness) specific to CALD communities, as well as other diverse groups. | *Diversity responsiveness*: Mental health services take into account the cultural and social diversity (including diversity in terms of religious/spiritual beliefs, gender, sexual orientation, age and socioeconomic status) of its consumers and meets their needs throughout all phases of care, |
| NSW Plan for Healthy Culturally and Linguistically Diverse Communities (2019-2023), NSW Health | 2019 | Healthcare services in general. Builds on the previous plan and in response to obligations under the *Multicultural NSW Act 2000.* | CALD community | Identify four outcome areas for NSW Health:   1. Strategies are in place to improve access and quality of care for CALD communities 2. Support for individuals from CALD communities to build health literacy 3. Responsive to people’s needs, language and culture 4. Understands the needs, experiences and identities of CALD communities | A culturally responsive health system values cultural and linguistic diversity and cultural knowledge, undertakes cultural self-assessment and provides appropriate language services and supports. Cultural responsiveness emphasises the capacity to respond to the healthcare issues of consumers. |
| A Strategic Framework for Action: Advice to the Australian Government on the Development of the Australian National Diabetes Strategy (2016-2020), Australian Health Associates | 2015 | Healthcare services in general, but specific to diabetes care and management. In response to the development of the National Diabetes Strategy. | Australian population, in general | Identification of seven high-level goals. Goal five is specific to high-risk and vulnerable populations, including the CALD community. | Actions within goal five are tailored to the specific needs of CALD communities to ensure that person-centred, culturally safe care respects diverse languages, religious beliefs and cultural practices.  This involves the translation of consumer resources into appropriate language, enduring access to translation services, improving health literacy through the dissemination of culturally appropriate information and development of information packages. |
| Cultural Competency in health: A guide for policy, partnerships and participation, NHMRC | 2005 | Healthcare services, in general. In response to the need for recognising the inclusion of cultural issues in health care and to increase cultural competency. | CALD community | Outlines four interrelated domains of cultural competency: systemic, organisational, provisional, professional and individual. | - Systemic- the need for effective policies and procedures which support the active involvement of CALD communities. - Organisational- cultural competency is “valued as integral to core business”. - Professional- cultural competency is an important component in education and professional development. - Individual- identification of knowledge, attitudes and behaviours. Individual health professionals are supported to work in diverse communities. |
