# Supplementary material for: A critical interpretive synthesis of migrants’ experiences of the Australian health system
Source: Int J Equity Health. 2023 Jan 9;22:7. doi: 10.1186/s12939-022-01821-2 (PMC9827657; doi:10.1186/s12939-022-01821-2)
Supplement: Supplementary file 3 — Additional file 3. Development of critiques and synthesising argument. [file 12939_2022_1821_MOESM3_ESM.docx]

**Additional File 3: Development of critiques and synthesising argument**

In this CIS, we aimed to determined how the literature could better conceptualise migrants’ experiences of the Australian health system. This document serves as a guide to the development of the three critiques and the overall synthesising argument. Specifically, it aims to provide the empirical and theoretical evidence which formed the basis of our critique of the Australian scholarly literature. We provide an overview of each critique with reference to the empirical evidence, presented in tables. Then, we articulate our proposal of how researchers and policymakers could better conceptualise and understand migrants’ experiences of the Australian health system, in the form of a synthesising argument. Here, we present the empirical and theoretical evidence which underpins this synthesising argument.

**How are migrants’ experiences of the Australian health system conceptualised?**

We began this CIS by considering how the Australian literature conceptualises migrants’ experiences of the Australian health system. Our critique of the Australian scholarly literature, as well as State and Federal Government health policies aimed to identify what was overlooked or not accounted for in current inquiries into migrants’ care experiences. Sensitised by the theoretical literature, we present our critique of the Australian scholarly literature and policies along three broad lines - these represent the key findings of this CIS. These critiques are outlined below with reference to the supporting empirical evidence.

## **Critique 1: Reduction of the migrant patient to ‘a’ cultural identity**

Within the examined Australian empirical literature, as well as State and Federal Government health policies, the influence of culture on migrants’ experiences of the Australian health system was widely reported and discussed. Table 1 outlines some key examples of how the cultural values, beliefs, and the associated stigma of these beliefs, shaped various aspects of migrants’ care encounters. In articulating these influences, we noted that migrant patients were often homogenised and reduced to specific cultural groups. The evidence in Table 1 highlights how migrants’ decision to access healthcare, their need for confidentiality, or autonomy during the care encounter, was influenced by their “Asian”, “Arabic” or “Indian” culture. In these studies, there is an assumption that migrant patients view themselves as “members” of specific cultural groups and, in this way, adhere to the common practices and beliefs of these groups. As such, categorising migrant patients according to their assumed “cultural identity” has led to an inordinate focus on ‘a’ culture as the key factor shaping migrants’ experiences of the Australian health system. However, there were exceptions, with several studies recognising the limitations of such a culturalist approach. As the evidence in Table 2 demonstrates, these studies (the exceptions) stressed the importance of considering the “individuality” of migrant patients. Given the cultural framing of migrants’ care experiences, it was therefore unsurprising that Australian policies on migrant health were also framed within a narrow cultural lens. As demonstrated in Table 1, multiple Australian healthcare policies emphasised that culture, in particular, influences how individuals experience and manage health and illness. However, as the evidence in Table 3 outlines, some studies in fact highlighted the potential shortcomings of cultural competence/responsiveness. Given these findings, we argue that an inordinate focus on ‘a’ culture as the key factor shaping migrants’ experiences of the Australian health system inappropriately presents migrants as “culturally encapsulated”.

**Table 1.**

| Cultural influences on migrants’ care experiences | |
| --- | --- |
| Record | **Evidence** |
| Alzubaidi et al. (2015) | - There can be problematic aspects of “collectivistic Arabic cultures”; one is expected to share life situations with significant others and involve them in decision-making processes, including those related to health. |
| Blignault et al. (2008) | - In the Chinese culture, there is an emphasis on an individual’s “forbearing ability”– to overcome pain and problems on their own. This influences Chinese migrant patients’ need for confidentiality, as well as their hesitancy to seek professional help for their mental health concerns. |
| Hoang et al. (2009) | - Most Asian cultures can teach people to be unassertive and inhibited from childhood. Therefore, Asian women were often too reluctant or embarrassed to express their needs and preferences (such as cultural practices following childbirth). - Apart from having an inexperienced doctor, a Vietnamese woman’s culture may have contributed to the trauma she experienced from an incorrect procedure. Enduring pain in silence and maintaining self-control without complaint has been reported as a traditional value of the Vietnamese. |
| Mohan et al. (2006) | - Putting themselves last and thinking about other family members was seen as part of Indian culture and this influenced individuals’ timely access to health services. |
| Youssef & Deane (2006), Saleh et al. (2012) | - For Arab migrants, the cultural context of diseases, such as cancer and diabetes, can be one where there is “no solution”, is seen as a “death sentence” and is therefore in the “hands of God”. This influenced healthcare access and utilisation. |
| Shaw et al. (2015) | - Individuals from the Arabic groups generally held the view that family should make decisions on behalf of the patient, relieving the patient of the burden of having to weigh up options. |
| Stanzel et al. (2020) | - Vietnamese migrant women were hesitant to discuss or ask for information related to menopause as, in their culture, health professionals were greatly respected and should initiate this conversation themselves. |
| Culturally and Linguistically Diverse Children and their families – Implications for paediatric and child development services in Queensland, Queensland Health (2019) | - In this report, culture is seen as a “dynamic yet stable set of beliefs and attitudes shared by a group of people”. Health, illness and disability are all concepts shaped by cultural values and beliefs and therefore culture will influence how we perceive, experience and manage health and illness. |
| Exploratory Analysis of Barriers to Palliative Care:  Issues Report on People from Culturally and Linguistically Diverse Backgrounds, Australian Government Department of Health (2019) | - The report recognises that CALD populations are diverse, with differences in language, cultural norms and religious beliefs. However, “culture plays a critical role in how patients, families, and healthcare providers view the end-of-life”. - “Collectivist” cultures (including many Eastern, Middle Eastern, African, South American and some Mediterranean cultures) tend to define themselves in terms of their relationships rather than individual characteristics. One’s identity is based on their social network. |
| NSW Plan for Healthy Culturally and Linguistically Diverse Communities (2019-2023), NSW Health (2019) | - Within the Plan, culture is reported to influence “how consumers define health and illness and how they perceive and respond to health information”. It also affects how “healthcare is sought, how symptoms are described, how treatment options are considered, and whether treatment will be chosen and adhered to” - Cultural beliefs and expectations, and lack of proficiency in English can lead to issues with accessing mainstream health services. |

**Table 2.**

| The “individuality” of the migrant patient | |
| --- | --- |
| Record | **Evidence** |
| Botfield et al. (2018) | - Young migrants did not appear to think of themselves as particularly unique or different from other young people living in Australia. - Many described their experiences as those of a ‘young person’, or being from the ‘younger generation’, rather than as someone from a particular cultural heritage or background. - Some young migrants did not identify with a particular cultural group or community, and those that did, did not always subscribe to the same beliefs or values as that group or community. - Therefore, there are intergenerational differences in attitudes and beliefs towards SRH issues and there is a need to consider “individuality”, particularly in healthcare interactions with young migrants. |
| Broom et al. (2019) | - Some health professionals sought to first understand the cultural expectations associated with the patients’ cultural background and whether they, as an individual, subscribed to them. Here, the health professional stresses the importance of individuation and the uniqueness of each patient - Health professionals also discussed managing the “reduction of personhood to cultural abstractions”; in some cases, family values and individual idiosyncrasies may inform decision making more than cultural practices - *“Because it’s part of the mix. I mean, you’re presenting that as though it’s a dichotomy, but it’s not a dichotomy. It’s just part of the complexity, and if the cultural aspect of that person is more predominant, then that’s going to be more of that complexity for that person. If there are other things that come into the mix and if you’ve got low SES, isolation, depression, and all that sort of thing, then you’re taking that all into account. So I don’t know that it’s an either/or kind of thing. I think it’s definitely important, but it’s not always the most important thing.”* |
| Johnstone et al. (2017) | - Not all participants believed that culture per se had a significant bearing on how patients and their loved ones experienced EOL care. Some participants strongly believed *“the culture doesn’t come into it”*. - While accepting that cultural considerations were important, one participant strongly believed that these only came into play after the patient had died, asserting, *“I don’t really decide based on culture. I just act on symptoms [ . . .]. It’s only after the patient passes away that you take this culture into consideration”.* |
| Renzaho & Oldroyd (2014) | - Migrant women could reconcile both sets of childbirth and pregnancy practices and did not describe these differences as a source of signiﬁcant conﬂict. Mothers described weighing both options (i.e. picking and choosing) which traditional cultural practices they would retain and which ones they would ignore. - *“…. yeah, actually my parents say you don’t have to listen too much to the [Australian] nurse because they [nurses] don’t really know about our Chinese traditional but for me I believe in Australia I take what the nurse said so I listen half and half…”* |

**Table 3.**

| Shortcomings of cultural competence/responsiveness | |
| --- | --- |
| Record | **Evidence** |
| Abdelmessih et al. (2019) | - The study results shed light on how cultural sensitivity appeared to be rejected by “Arabic-speaking” patients due to fear of being stereotyped. They perceived consideration of their cultural/religious beliefs, and being provided with tailored health information, as being regarded as diﬀerent (i.e., not being regarded as members of the Australian society). Participants did not prefer to be regarded as diﬀerent and preferred to be viewed as integrated members of the Australian society to avoid racism, which some have reported experiencing. |
| Broom et al. (2019) | - There is a Catch-22 of cultural awareness; health professionals should learn about and, be sensitive to the cultural values of the patient, but this could lead to a lack of recognition of the individuality of each patient. - *“In very complex care scenarios, you almost need people who subspecialise in (cultural competence) in a way because it’s almost too much to assume everyone maintains the competency you need to have to look after these patients, in a way. There needs to be a group of people who have quick resources, the extra time, and they’re actually protected from having to treat them in a quick way. That’s the only way it’s going to be done properly”.* |
| Johnstone et al. (2016) | - Healthcare providers discussed the challenge of not making assumptions about what is “culturally appropriate” as patients may not always follow certain cultural beliefs. |
| Johnstone et al. (2017) | - Most health professionals had little knowledge or understanding of what constituted culturally responsive EOL care. They felt overwhelmed when first encountering patients and families from diverse cultural backgrounds. - Healthcare professionals’ main source of learning was *“on the job”.* |
| Mengesha et al. (2018) | - Cross-cultural training, where it exists in the health system, focused on Indigenous Aboriginal health with little attention to refugee and migrant health. - *“There is now a huge push for us to learn about the Aboriginal culture and it’s become mandatory that we do some of the training on that. But there’s nothing really to—along similar lines to educate us about these other cultures and about some of the traumas and such that people go through.”.* |
| Mollah et al. (2018) | - Two practitioners that disagreed about culturally competent care said that the imperative to be culturally competent risked generalisation and stereotyping and, as a result, rejected the need to be culturally competent at all. - *“I’ve come across too many cultures to be competent in all of them… so I tend to… go cautiously, be reserved to begin with… follow their initiative, so if they’re happy to shake hands, great, I’m happy to shake hands… just being open to it, and not being offended.”.* |
| Truong et al. (2017) | - Though aware of the term “cultural competence”, some healthcare professionals struggled to describe their understanding of the concept. - One healthcare professional suggested treating people with universal values such as ‘respect’ and ‘dignity’ regardless of cultural background. She felt that she could not be expected to know everything about every culture and that it was not necessarily relevant for every client. |
| Wamwayi et al. (2019) | - Healthcare professionals felt ill-equipped to provide culturally appropriate care due to lack of cultural awareness training. - *“The only tool I have is that I am African, that is all the tool I have but I have no training.”.* |

## **Critique 2: Reduction to and of language**

Another consistent finding within the Australian literature was that migrants’ care experiences were shaped and defined by language and communication-related barriers. Studies overwhelmingly reported that migrants, who were unable to speak and understand English, could not comprehend and/or effectively communicate their needs to healthcare practitioners, as well as understand English-based education resources. Given the significant impact of language on the migrant care experience, a number of studies specifically examined the influence of language and communication-related barriers. Such an articulation of language and communication barriers experienced by migrant patients is expectedly a dominant feature in the Australian scholarly and policy literature. Moreover, as is evident in Appendix 1, multiple studies categorised migrants according to specific language groups labelling migrants as, for example, “Arabic speaking” or “Chinese speaking”. We argue that the reduction of migrants to specific language groups in these studies inappropriately conveys a sense of “linguistic homogeneity”. That said, some studies did note the importance of recognising the heterogeneity *within* language groups and this evidence is outlined in Table 4. Drawing on this evidence, we argue that reducing migrants to their language group is problematic as it prejudicially anticipates their English-language ability and discounts the diversity within migrant populations. In doing so, migrants’ experiences of the Australian health system are defined by the inevitable language barriers they encounter.

**Table 4.**

| Reduction of language | |
| --- | --- |
| Record | **Evidence** |
| Alananzeh et al. (2018) | - Participants emphasised the need to use interpreters who spoke the same Arabic dialect. Using interpreters with different dialects resulted in not being able to understand what the interpreter was trying to say or understanding some words incorrectly. - *“There was a problem with translation. The translator there was from Lebanon and I am Egyptian, and there are many differences between the two dialects. At first, the translator was a Lebanese lady, but I asked them to change her, I wanted an Egyptian one. I can’t understand her, for example, “badana” mean “start” for Lebanese people but for Egyptian mean “finished””.* - *“With the Egyptian translator, I had a problem in the dialect. Yes, I couldn’t understand everything. The Egyptian dialect is hard to understand. Yes, you know it’s very hard to understand Egyptian dialect. I can understand you (Jordanian interviewer) for example, nevertheless you are not a translator but your dialect is understandable”.* - As a result of the different dialects and nuances, some participants felt that it was hard for the interpreter to convey the meaning of the conversation, resulting in incorrect translation. |
| Blignault et al. (2008) | - Participants found that there were insufﬁcient Mandarin-speaking mental health professionals, in both the public and private sectors. Several commented that professional interpreters were not always available when required. - One participant described the ‘circus’ that resulted after she organised for a Cantonese-speaking bilingual worker to accompany her to the patient’s home and, on arrival, discovered the patient spoke Mandarin. In the end, they had to rely on a telephone interpreter for communication. |
| Botfield et al. (2017) | - Several participants identified challenges that extended beyond language itself, suggesting there were deeper issues related to nuances within language, including the issue of original meanings being ‘lost’ or ‘changed’ during interpretation and translation. - One commented that *“there is the reality that sex is often conceived differently, and sexual health is often conceived differently, across cultures, so there may be a challenge in the language that [clinicians] use”.* |
| Henderson & Kendall (2011) | - Community navigators explained that within their own cultures, there were different gender-based norms and beliefs, religious groups and language clusters. Despite having a cultural background, participants were not always cognisant of the speciﬁc requirements of sub-groups within their community. |
| Wamwayi et al. (2019) | - Participants were of the view that most of the information provided to African patients was in English and therefore culturally inappropriate. They believed health information should be available in many different languages inclusive of the major African languages. |

## **Critique 3: Homogenisation and reduction of migrants’ geographic origins**

Apart from categorising migrants according to their language group or “cultural identity”, some studies homogenised and reduced migrants to broad geographic locations. As Appendix 1 reveals, a number of studies categorised migrants according to their geographic origins, such as Sub-Saharan Africa or South-East Asia, in order to compare the commonalities and differences in the care experience. In doing so, these studies assume that migrants originate from distinctly bounded, homogenous societies and therefore do not specifically engage with the possible differences *within* these groups. Though much of the literature did not appreciate the problems associated with such homogenisation and reduction, Table 5 highlights the valuable insights some studies offered on how this can be problematic. In light of the evidence, we argue that homogenising migrants and reducing them to broad geographies ignores the heterogeneity within and across countries and regions, as well as the subsequent impact such diversity can have on migrants’ experiences of the Australian health system.

**Table 5.**

| Heterogeneity within migrant groups | |
| --- | --- |
| Record | **Evidence** |
| Abdelmessih et al. (2019) | - Almost all Arabic-speaking participants identiﬁed that they do not need written information that is tailored to their cultural needs as individuals’ cultural needs vary. Diﬀerent Arabic countries have slightly diﬀerent cultures, and diﬀerent parts of the same country may have slightly diﬀerent cultures. |
| Cross and Bloomer (2010) | - Individual differences should be noted and the delivery of holistic care maintained. Health professionals in this study realised that, within any culture, there is individual difference and that it was easy to get trapped into using cultural stereotypes rather than working with individual differences. |
| Henderson & Kendall (2011) | - Community navigators explained that within their own cultures, there were different gender-based norms and beliefs, religious groups and language clusters. Despite having a cultural background, participants were not always cognisant of the speciﬁc requirements of sub-groups within their community. |
| Wamwayi et al. (2019) | - Healthcare providers found it difﬁcult to understand individual patients’ cultural background due to language barriers where cultural differences between different ethnic African groups equates with different beliefs and healthcare practices and understandings. |

**How could migrants’ interactions with and experiences of the Australian health system be conceptualised?**

Our critique of the Australian literature aimed to highlight the gaps in how the migrant care experience has been conceptualised. Below, in the form of a synthesising argument, we outline our proposal of how the literature could better conceptualise and understand migrants’ experiences of the Australian health system. In Table 6, we highlight the empirical evidence which forms the basis of this argument and the links to the relevant theoretical literature.

## **Synthesising argument**

The Australian scholarly literature, and State and Federal Government health policies frequently categorise migrants according to an assumed “cultural identity”, linguistic affiliation and/or geographic origin. We argue that such a reduction and homogenisation problematically presents migrants as “culturally encapsulated”, prejudicially anticipates their English-language ability and, ultimately, discounts the diversity and heterogeneity within migrant populations in Australia. This reduction and homogenisation are consequently reflected in a narrow policy focus on how culture and language define migrants’ experiences of the Australian health system. Instead, we propose that future Australian and international research examining migrants’ care encounters should consider the multiplicity and fluidity of migrant identities. As outlined in Table 6, the studies by Broom et al. (2020), Maharaj and Bandyopadhyay (2013), and Maneze et al. (2018) revealed the multiple and fluid identities that migrants assumed during their encounters with the health system. However, other studies also demonstrate how these identities are socially constructed and can be reinforced and contested relationally, during migrants’ interactions with health professionals and their “counter-identities”. We argue that these understandings have important implications for the implementation of Australian health policies and programs for enhancing health systems responsiveness to the needs and expectations of migrants.

**Table 6.**

| The multiplicity and fluidity of migrant identities | | |
| --- | --- | --- |
| Record | **Evidence** | **Links to theoretical literature** |
| Abdelmessih et al. (2019) | - One participant recalled that her culture was mocked by health providers in hospital, which resulted in her family’s poor trust in the health care system, and their reluctance to seek health care. *“Staﬀ [hospital staﬀ] made fun of my culture there . . . so we [including family members] do not want to go back there.”.* - “Arabic-speaking” patients believed that consideration of their cultural/religious beliefs, and being provided with tailored health information, as being regarded as diﬀerent (i.e., not being regarded as members of the Australian society). Participants did not prefer to be regarded as diﬀerent and preferred to be viewed as integrated members of the Australian society to avoid racism, which some have reported experiencing. | **Relationality**   - Migrant identities are socially constructed constituted relationally during their interactions with health professionals and their “counter-identities” (Carter and Mireles 2015, Hack-Polay et al. 2021, Li and Findlay 1996, McAreavey 2017).   **Multiplicity of migrants’ identities**   - In order to navigate the new social and cultural landscape, migrants’ may suspend their “native” selves, including their cultural beliefs, practices, as well as language. They may be pressured to do so by the social system and institutional structures to avoid racism or discrimination (Hack-Polay et al. 2021). |
| Broom et al. (2020) | - The sense of “intruder status” can be profound and can shape experiences of care. Patients read therapeutic relations and exchanges as often being underpinned by ideas about race/ethnicity (e.g., “non-White”), identity politics (e.g., “Australian”), and a discomfort therein (on the part of service providers and themselves). - This reflected intersections between public health care, assumed costs of being non-White/foreign, or assumptions about cultural groups (e.g., literacy). - *“You go and [as a migrant] you’re almost an intruder into their space. <laughter> Just about anywhere you go in the medical system, you walk and you speak to the receptionist and a lot of times you’re infringing on their time…. She [receptionist] was responding to not being Australian, not being white. She assumed that I was not going to be able to fend for myself and the language.”* - As outlined across both patient and professional experiences, migrants can be asked to be paradoxical things. As new citizens, they are entitled to the same care, but should not feel entitled; should be assertive as the new health system demands it, but should also be aware of not coming across as ungrateful. For example, there were recurrent references throughout the study’s focus groups about being “grateful”. | **Relationality**   - Migrants’ multiple identities of “non-White”, “foreign” and “intruder” can be reinforced and contested relationality. These identities were validated externally during their interactions with health professionals and other health system actors and their “counter-identities” (Carter and Mireles 2015, Hack-Polay et al. 2021, Li and Findlay 1996, McAreavey 2017).   **Multiplicity of migrants’ identities**   - Migrants may be caught between contradicting and evolving constructions of identity. In this case, migrants needed to be assertive during their encounters with health professionals, while also trying not to appear “ungrateful” (Hack-Polay et al. 2021, Li and Findlay 1996, McAreavey 2017). |
| Benza & Liamputtong (2017) | - Women described being treated without respect and being discriminated against. Some also reported being labelled and receiving racially stereotypical comments when attending the healthcare system. - *“I think it's hard and people already have these thoughts about you as you walk through the door, like as they see this African woman, they think oh she's here to cause trouble. I even heard another midwife saying “She should be happy that she's in Australia”* | **Relationality and “significant symbols”**   - One’s appearance as an “African woman” can be a “significant symbol” which health professionals may subconsciously judge and respond to with, in this case, racially stereotypical comments (Carter and Mireles 2015). |
| Butow et al. (2010) | - Some participants felt judged to some degree as different or alien. *“…the doctor…[if] he reckons you won’t do much good to the society or he thinks you are not significant… He judges your value to decide if he should save your life… The doctor sees if you can speak English, if not, you are a handicapped person”.* | **Relationality and “significant symbols”**   - A health professional may subconsciously judge a patient’s ability to speak English which can be “significant symbol”. In this example, their subsequent response may have reinforced this migrant patient’s sense of being ‘othered’ and being seen as “different” or “alien”. |
| Maharaj and Bandyopadhyay (2013) | - One woman migrated from India over ten years ago and felt that she was acculturating to an Australian way of life. Gaining a sense of belonging meant adopting Australian gender norms. Apart from needing to work to maintain a good standard of living, she wanted to work as she felt this made her independent and gave her an identity, enablingher to embrace roles apart from ‘mother’. Acculturating women were dealing with a shift in personal identity from the traditional Indian mother to incorporating a more Westernised gender roles. - Thus, women were balancing two distinct models or cultural approaches to motherhood: the Western or Australian model (individualistic or self-sufficient approach) in which women are generally expected to cope on their own and the traditional or South Asian approach to mothering, dominated by a cultural expectation that the new mother be supported and nurtured. Women who were in Australia longer, worked and followed the Western approach to motherhood. | **Fluidity and multiplicity of migrants’ identities**   - Migrants define themselves differently across place and time and along the various phases of the migratory process (Li and Findlay 1996). - They may also suspend their “native” selves, including their traditional cultural values and beliefs related to pregnancy and motherhood. As a result, migrant women may assume the multiple and shifting identities and roles of “mother” and “professional”, in order to navigate the new social and cultural landscape (Hack-Polay et al. 2021). |
| Maneze et al. (2016) | - Some of the participants expressed their belief that health professionals did not listen to what they were saying in English but instead were judging their language ability by their appearance. - *“Sometimes I feel like, you know, I am talking to you in English, you look at me because I am an Asian and if I speak in English, you don’t understand me because you are not listening to what I say but you look at me because I am an Asian. Could be the doctor or in the shops, I am speaking to you in English, I don’t understand why you still can’t understand what I say. You look at me but you don’t listen to what I say, then I speak English slowly, then I think maybe he is not listening to me because of the way I look.”* | **Relationality and “significant symbols”**   - An individual’s inability to speak English, as well as their “Asian” appearance, can be “significant symbols” which health professionals may subconsciously judge and respond to, and, inadvertently reinforce a sense of being ‘othered' during the care encounter (Carter and Mireles 2015, Pedwell 2010). |
| Maneze et al. (2018) | - Some participants felt that they were ‘visitors’ in Australia and as recipients of this hospitality they had to be ‘grateful’. - Participants disclosed their reticence and embarrassment to challenge health providers’ orders, who they considered to be experts; therefore, their views were not to be challenged. *“Here (in Australia), sometimes you are embarrassed or reticent (nahihiya) to tell the doctor how you feel especially when you have to say it in English. Unlike in the Philippines, you can tell the doctor how you really feel deep in your heart. Here sometimes I don’t feel like I can do that.”.* | **Fluidity and multiplicity of migrants’ identities**   - Migrants may be caught between contradicting and evolving constructions of identity, across time and place. As opposed to a care encounter in the Philippines, migrants felt like “visitors” in Australia which shaped their inability to express their needs to health professionals. (Hack-Polay et al. 2021, Li and Findlay 1996, McAreavey 2017). |
| Raymundo et al. (2021) | - Some participants also described experiences of their concerns being dismissed by health professionals. *“I think the thing that stuck out to me the most was when she said, ‘You should just be happy that you live in Australia.’ So after I talked about my problems, or my feelings, that's what she said to comfort me, and I don't feel like that was acceptable to any degree.”.* | **Relationality**   - One’s identity as a “migrant” and their need to be “grateful” can be constituted relationality, during their interactions with health professionals (Carter and Mireles 2015, Hack-Polay et al. 2021, Li and Findlay 1996). |
| Shafiei et al. (2015) | - One woman mentioned health professionals' attitudes towards Muslims as a barrier to seeking help*: “When you're a Muslim and you wear [a] scarf, they think and feel like you don't need or you don't deserve to get help the same way as an Australian or someone who is not Muslim … that makes you feel like I don't want to tell her anything about my problems. What for, she doesn't even like me.”.* | **Relationality and “significant symbols”**   - Health professionals may subconsciously judge a patient’s appearance which identifies them as a “migrant” and a Muslim. The health professional’s subsequent response to this “significant symbol” can inadvertently reinforce a migrant patient’s sense of being ‘othered' (Carter and Mireles 2015, Pedwell 2010). |
| Truong et al. (2017) | - A health professional described how she could empathise with clients from a migrant background that struggled to speak English as her parents also had a similar experience. *“I think if you’ve got experience, like I said I have, in terms of my parents being migrants...if you’ve got that type of thing you’re sort of more understanding. Maybe some people who work here don’t have that behind them and have found it a bit harder, a bit more difficult”.* | **Relationality**   - A health professional’s “counter-identity” can shape how they judge and respond to a patient’s migrant identity with, in this instance, empathy (Carter and Mireles 2015, Hack-Polay et al. 2021, Li and Findlay 1996). |
